# Competence of pharmacy mentors: a survey of the perceptions of pharmacy postgraduates and their mentors

**DOI:** 10.1186/s12909-020-02188-0

**Published:** 2020-08-12

**Authors:** Juan-Juan Yue, Gang Chen

**Affiliations:** 1 Department of Clinical Microbiology and Immunology, Faculty of Pharmacy and Medical Laboratory Sciences, Third Military Medical University (Army Medical University), Chongqing, 400038 China; 2 Biomedical Analysis Center, College of Basic Medicine, Third Military Medical University (Army Medical University), No. 30 Gaotanyan Street, Chongqing, 400038 People’s Republic of China

**Keywords:** Competence, Pharmacy, Mentor, Survey

## Abstract

**Background:**

Although mentorship can bring many benefits to medical education, mentors’ need for professional development is typically ignored. This study aims to acquire insight into the development of pharmacy mentors’ competence by comparing differences between mentors’ and postgraduates’ perspectives.

**Methods:**

We used ANOVAs, independent-sample T-tests and paired-sample T-tests to analyze data collected via an anonymous survey, which included a prepared questionnaire completed by 118 pharmacy mentors and 118 pharmacy postgraduates from 8 Chinese universities and colleges.

**Results:**

1. Research competence, professional knowledge, and communication competence exhibited the highest means. 2. Research competence was highly correlated with communication competence and moderately correlated with professional knowledge, educational competence, academic achievement and supportive competence. 3. Mentors’ educational competence was significantly more important to mentors than to postgraduates, and mentors’ supportive competence was significantly more important to postgraduates than to mentors. 4. Educational competence, supportive competence and academic achievement were significantly more important to mentors with a bachelor’s degree than to mentors with a master’s or doctoral degree. 5. Research competence, educational competence and communication competence were significantly more important to female students than male students.

**Conclusions:**

Good mentors should possess three core competencies: research competence, professional knowledge and communication competence. They are related rather than independent. The construction of a harmonious mentoring relationship should take full account of a student’s characteristics and expectations because graduate students care more about supportive competence and female students assign greater importance to mentors’ competence than male students. There should be more development opportunities for less educated mentors, as they have a greater need to increase their competence than more qualified mentors.

## Background

Mentoring significantly influences pharmacy students’ professional development, career orientation, choice of career and research productivity, including success in publishing scientific articles and obtaining research grants [1–3]. Mentoring is also beneficial to mentors, as they may experience more job satisfaction and self-esteem by sharing knowledge with young students and having the opportunity to learn from mentees [4, 5]. Therefore, mentorship is widely applied in higher pharmaceutical education.

Although mentorship can bring many benefits to graduate education, most universities and educational research remain focused only on newly qualified teachers. As a result, relatively little is known about mentors’ professional knowledge and needs [6]. Therefore, mentor education needs to be developed and studied under certain circumstances to work effectively, as it is difficult to adopt a model for mentor education from another context [6].

There is no universal definition of mentoring. Mentoring can be performed in many contexts, with a variety of purposes and theoretical approaches [7], and under different circumstances in a variety of ways with different durations and intensities [8]. For example, in some situations, the terms ‘mentor’ and ‘mentor education’ are used in the context of ‘preservice education’ and focus on initial teacher training, student teachers and their mentors [6, 9, 10]. In this study, the nature of the mentor-mentee relationship is an extension of the teacher-student relationship [11]. The mentors in this study were experienced teachers who had been selected into pharmacy professional mentoring programs according to the standards of each Chinese university. In these programs, mentorships are formal, and these mentor-mentee pairs are arranged by the universities [12]. The mentoring takes place mainly during the academic careers of students [13], although it also exists in a variety of practice environments, such as the community, institutions, pharmacies and other practice settings [14]. The mentors need to support assigned postgraduates in their learning and are responsible for teaching, guiding and assessing students in professional practice for two to three years. The mentors have absolute authority regarding postgraduate curriculum, academic research, rewards and punishments, graduation and employment recommendations. The relationship between mentors and mentees differs from the general relationship between teachers and students [15].

Many factors affect the success of the mentor-mentee relationship, and it is difficult to construct a universal model suitable for any context [5]. Clearly, however, a focus on mentors’ competences is pivotal, as mentors are the main role models and experts guiding students [16]. Mentoring programs to develop professionalism among pharmacy students have often been presented in detail [17], but little is known about how mentors should prepare themselves for the successful implementation of these projects. The Accreditation Council of Pharmacy Education Guidelines lists the behaviors, qualities, and values suggested for preceptors; these behaviors, qualities, and values include behaving ethically and showing compassion for patients; accepting personal responsibility for patient outcomes; preceptors’ having professional training, experience, and competence commensurate with the preceptors’ position; and using clinical and scientific publications in clinical care decision-making and evidence-based practice [18]. However, the guidelines fail to provide other suggestions, such as which of these behaviors or qualities is more important? Currently, clinical pharmacists and academic researchers are the two main types of pharmacy postgraduate careers cultivated in China. Whether in a clinical or an academic setting, mentorship refers to a complex and multidimensional process [19] that integrates individual and organizational aspects and environmental, collegial, pedagogical and clinical attributes [20]. However, there are few studies about pharmacy mentors’ competence in China.

In our previous study, we built the theoretical framework for Chinese pharmacy mentors’ competence, including research competence (evaluated on 11 items), educational competence (evaluated on 6 items), supportive competence (evaluated on 5 items), communication competence (evaluated on 5 items, academic achievement (evaluated on 5 items), and professional knowledge (evaluated on 5 items). There are six dimensions with 37 items, which explain 67.12% of the total variance [15]. This study explores the relationship among the six competences further by investigating and analyzing differences between the perspectives of mentors and postgraduates regarding pharmacy mentors’ competences in Chinese universities. We aim to study mentor and student expectations to identify the profiles of good mentors’ competences to promote understanding and cultivation of mentors’ professional competence.

## Methods

### Participants and data collection

This pilot survey involved a general sample of 118 pharmacy professional master’s degree mentors and 118 pharmacy professional degree postgraduates from eight Chinese universities and colleges. The pharmacy professional mentors and postgraduates were all volunteers.

The study was approved by the Institutional Review Board at Army Medical University. All experiments were performed in accordance with relevant guidelines and regulations.

### Instruments and measurements

1. The demographic information collected for the mentors included age, sex, professional title, educational background, teaching experience and units, and the demographics collected for the postgraduate students included sex, grade and units. Detailed information was presented in our earlier study [15].
2. A questionnaire called ‘Research on the competence of pharmacy professional mentors in Chinese universities’ was developed in our earlier study [15]. The questionnaire consisted of 37 items in 6 categories: research competence, educational competence, supportive competence, communication competence, academic achievement and professional knowledge. We used a 7-point Likert scale (the score for each item ranged from 1 to 7, 1 = not important, 7 = very important) [21]. A high score indicated strong competence. The questionnaire’s overall reliability and all reliability dimensions of the self-rating scale were good, with an overall Cronbach’s alpha coefficient of 0.957, and the alpha values ranged from 0.831 to 0.921 for the six subscales.

### Procedures

The investigative procedure was described in our earlier study [15]. Briefly, we conducted an anonymous survey with the prepared questionnaire and polled 118 pharmacy mentors and 118 pharmacy postgraduates from 8 Chinese universities and colleges in Xian, Chongqing, Kunming and Chengdu cities. A total of 225 questionnaires were returned for a response rate of 95%. Any questionnaire with less than 95% completion or with the same score for each option was excluded, and nine unqualified questionnaires were eliminated. Therefore, the effective sampling number was 216, with an effective rate of 91%.

### Data analysis

The scores for the items in each category were summed to generate a score for the category, such as research competence, educational competence, supportive competence, communication competence, academic achievement and professional knowledge. The total score for each mentor was aggregated from the scores for all six categories. The data were analyzed using SPSS 13.0. The mean of six competences was analyzed using descriptive statistics on data provided by 216 respondents. Product-moment correlation analysis was used on these data to determine the relationships among the six subscales. ANOVAs were used on the six subscales and the overall score to test for differences among three or more groups, such as mentors classified by age, teaching experience and professional title. Independent-sample T-tests were used on the six subscales to test differences in perceptions of mentors’ competences between two groups, such as mentors and postgraduates, male and female. Paired-sample T-tests were used on data provided by 216 respondents to test differences of the six subscales, as it is unreasonable to conclude which competence is the most important by comparing the mean of the six competences without performing a difference test on the means [22], and *p* < 0.05 was considered to indicate statistical significance.

## Results

### The correlations and differences among the six competences

The results of the product-moment correlation analysis showed that there were significant positive correlations among the six competences (*p* < 0.01) (Table 1). Research competence was highly correlated with communication competence (*r* = 0.737, *p* = 0.000), thus indicating that the survey respondents thought that the stronger the communication ability of the mentors was, the stronger their research ability. Research competence was also moderately associated with professional knowledge, educational competence, academic achievement and supportive competence (0.4 ≤ *r* ≤ 0.7, *p* < 0.01).

**Table 1:** Correlation matrix of the 6 competences(*n* = 216)

	Research competence	Educational competence	Supportive competence	Communication competence	Academic achievement	Professional knowledge
Research competence	1					
Educational competence	0.621^**^ (R^2^ = 0.386)	1				
Supportive competence	0.444^**^ (R^2^ = 0.197)	0.538^**^ (R^2^ = 0.289)	1			
Communication competence	0.737^**^ (R^2^ = 0.543)	0.590^**^ (R^2^ = 0.348)	0.464^**^ (R^2^ = 0.215)	1		
Academic achievement	0.559^**^ (R^2^ = 0.312)	0.636^**^ (R^2^ = 0.404)	0.514^**^ (R^2^ = 0.264)	0.519^**^ (R^2^ = 0.269)	1	
Professional knowledge	0.658^**^ (R^2^ = 0.433)	0.575^**^ (R^2^ = 0.331)	0.465^**^ (R^2^ = 0.216)	0.594^**^ (R^2^ = 0.353)	0.587^**^ (R^2^ = 0.345)	1

** *p* < 0.01

The highest mean was found for research competence (m = 5.78, sd = 0.87), followed by professional knowledge (m = 5.72, sd = 1.01), communication competence (m = 5.65, sd = 0.96), supportive competence (m = 5.20, sd = 1.19), educational competence (m = 4.92, sd = 1.12), and academic achievement (m = 4.77, sd = 1.21). The results of the paired-sample T-tests (Table 2) showed that there were no significant differences between research competence and professional knowledge or between professional knowledge and communication competence. There were significant differences between communication competence and supportive competence (*p* = 0.000), supportive competence and educational competence (*p* = 0.000), and educational competence and academic achievement (*p* = 0.025).

**Table 2:** Paired-sample T-test for the 6 competences (*n* = 216)

	Mean ± SD	Std.Error Mean	t	P
Pair1 Research competence& Professional knowledge	0.059 ± 0.787	0.054	1.093	0.276
Pair2 Communication competence & Professional knowledge	−0.068 ± 0.885	0.060	−1.122	0.263
Pair3 Supportive competence & Communication competence	−0.454 ± 1.130	0.077	−5.899	0.000
Pair4 Educational competence & Supportive competence	−0.277 ± 1.115	0.076	−3.654	0.000
Pair5 Educational competence & Academic achievement	0.153 ± 0.999	0.068	2.255	0.025

### Evaluation of the differences between mentors and postgraduates

Mentors and postgraduates had significantly different views about educational competence and supportive competence (Table 3). The educational competence of mentors was significantly more important to mentors than to postgraduates, while the supportive competence of mentors was significantly more important to postgraduates than to mentors. There were no significant differences for research competence, communication competence, academic achievement or professional knowledge.

**Table 3:** Differences between mentors and postgraduates regarding pharmacy mentors’ competence

Subscale	Mentors (***n*** = 108)	Postgraduates (***n*** = 108)	T	p
Research competence	64.796 ± 7.101	62.278 ± 11.431	1.945	0.053
Educational competence	30.815 ± 5.554	28.213 ± 7.557	2.883	0.004
Supportive competence	24.639 ± 5.623	27.324 ± 6.014	−3.389	0.001
Communication competence	28.630 ± 3.909	27.870 ± 5.516	1.167	0.245
Academic achievement	23.833 ± 5.666	23.824 ± 6.433	0.011	0.991
Professional knowledge	28.769 ± 4.765	28.407 ± 5.307	0.526	0.599

Values are expressed as the means of three replicates with standard deviations (Mean ± SD).

**Fig. 1:**
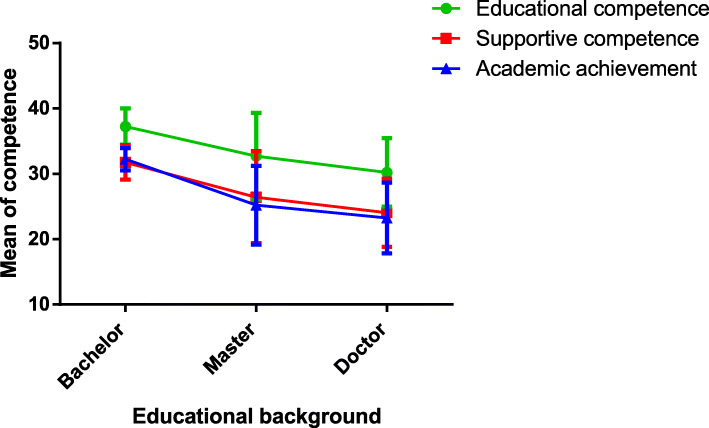
Means of three different competences of mentors with different educational backgrounds

There were significant differences among mentors with different backgrounds regarding the importance of educational competence, supportive competence and academic achievement (Table 4). Mentors’ educational competence, supportive competence and academic achievement were significantly more important for mentors with a bachelor’s degree than for mentors with a master’s degree and mentors with a doctoral degree. As mentors’ educational background increased, the sense of the importance of the three competences gradually decreased (Fig. 1). There were no significant differences in the importance of research competence, educational competence, supportive competence, communication competence, academic achievement, professional knowledge, supportive competence, academic achievement of professional knowledge for mentors of different sexes, ages, teaching experience and professional titles.

**Table 4:** Importance of pharmacy mentors’ competence for mentors from different educational backgrounds

Subscale	Mentors’ educational background			F	P
	Bachelor’s(*n* = 4)	Master’s(*n* = 14)	Doctorate(*n* = 90)		
Research competence	70.000 ± 8.756	64.710 ± 7.237	64.580 ± 7.009	1.120	0.330
Educational competence	37.250 ± 2.754	32.714 ± 6.650	30.233 ± 5.253	4.239	0.017
Supportive competence	31.750 ± 2.630	26.429 ± 7.046	24.044 ± 5.234	4.717	0.011
Communication competence	31.500 ± 2.380	28.714 ± 3.384	28.489 ± 4.015	1.143	0.323
Academic achievement	32.250 ± 1.708	25.214 ± 6.015	23.244 ± 5.420	5.791	0.004
Professional knowledge	31.750 ± 1.500	28.714 ± 5.150	28.644 ± 4.788	0.812	0.447

Values are expressed as the means of three replicates with standard deviations (Mean ± SD).

Postgraduates had significantly different views about research competence, educational competence and communication competence according to sex (Table 5). The importance of these competences was significantly higher for female students than for male students. There were no significant differences for supportive competence, academic achievement, or professional knowledge.

**Table 5:** Postgraduates’ perceptions of the importance of pharmacy mentors’ competence according to postgraduates’ sex

Subscale	Postgraduates’ sex		t	p
	Male (*n* = 41)	Female (*n* = 67)		
Research competence	58.830 ± 15.417	64.388 ± 7.475	−2.159	0.036
Educational competence	26.244 ± 8.532	29.418 ± 6.677	−2.154	0.034
Supportive competence	26.707 ± 6.112	27.702 ± 5.967	−0.833	0.407
Communication competence	26.317 ± 6.904	28.821 ± 4.246	−2.092	0.041
Academic achievement	23.537 ± 7.078	24.000 ± 6.053	−0.362	0.718
Professional knowledge	27.293 ± 6.396	29.090 ± 4.430	−1.582	0.119

Values are expressed as the means of three replicates with standard deviations (Mean ± SD).

## Discussion

Research competence, professional knowledge and communication competence were recognized as the most important competences of pharmacy mentors.

Academic research is an important part of postgraduate training. The Accreditation Council of Pharmacy Education Guidelines (Guidelines 23.4) states, ‘colleges and schools should implement strategies and programs to broaden the professional horizons of students in areas such as scientific inquiry, scholarly concern for the profession, and the relevance and value of research’ [18]. Therefore, research competence is always considered the core competence of health science teachers [23], and the quantity and quality of science papers published and national research topics are important evaluation indices for induction and on-the-job evaluations of mentors in Chinese universities, such as Tsinghua University and Peking University [24]. Meanwhile, research competence could stimulate the self-education and professional development of teachers and their ability to comprehend new ideas to be implemented for educational purposes [25]. In the study, mentors’ research competence was significantly positively correlated with educational competence (*r* = 0.621, *p* < 0.01). Like Burke-Smalley et al. [26] argued that the ability to successfully integrate research and teaching is the essence of a university professor. In general, mentors should strengthen their comprehensive abilities, especially their research ability, in postgraduate education [27].

The transmission of knowledge and information from the mentor to the mentee is an integral part of mentoring [5]. Teachers’ subject matter knowledge and pedagogical content knowledge have been argued to be essential to educational quality [28, 29]. Pharmacy, which is based on chemistry, biology, and medicine, is a highly professional and practical discipline that has a very complex knowledge system [14, 30]. The Accreditation Council of Pharmacy Education Standards (Standards 20) state that ‘mentors should have professional credentials and expertise commensurate with their responsibilities to the professional program [18]. Additionally, the study indicated that professional knowledge was positively correlated with the other five competences. The correlation with research competence was the highest (*r* = 0.658, *p* < 0.01). Professional knowledge is an indispensable component of creativity [31, 32], which is the basis for mentors’ scientific research. Therefore, mentors should continue to acquire professional knowledge, such as of pharmaceutics, clinical medicine, biology, chemistry, new experimental technology and database search methods. Mentors should also continue to gain practical experience in long-term pharmacy-related work and to participate in pharmacy, hospital and pharmacy practice every year to update their knowledge to provide better guidance.

Teacher communication skills are necessary for improving student learning. Mainhard et al. [33] reported that teachers can be most effective when they convey relatively high levels of interpersonal agency and communion in class. Bargar and Duncan [34] advised academic supervisors of postgraduates to follow several principles to promote students’ creative dissertation work, including building a friendly and equal relationship between teachers and students, thereby facilitating good communication. Furthermore, we found that mentors’ research competence and communication competence were highly correlated (r = 0.737, *p* < 0.01), and communication competence explained 54.3% of the total variance in research competence. Communication led to higher levels of team cooperation [35] and more support and resources [36, 37], which play important roles in research ability. Therefore, we should strengthen mentors’ communication skills training through lab meetings, teaching discussions, speech contests and language expression training courses to improve mentors’ professional development and students’ learning.

The perceptions of postgraduates and mentors with regard to mentors’ competence differed. Mentors thought educational competence was more important to mentors’ competence development and were not concerned about educational competence to the same degree as postgraduates. Mentors’ educational competence determines the quality of classroom teaching and their effectiveness in guiding students. The core content of this competence is to foster effective teaching behavior, such as creating a safe and stimulating learning climate, employing efficient classroom management strategies, providing clear instruction, activating learning, and adapting teaching and teaching-learning strategies [38–40]. Mentors want opportunities to participate in professional development programs to improve their teaching ability and become better prepared for mentoring. However, postgraduates may lack a comprehensive understanding of mentors’ competence and may think that increasing mentors’ educational competence does not help postgraduates’ personal growth, especially with regard to earning a graduate degree.

The findings suggest that postgraduates placed more importance on supportive competence than did mentors and that mentors cared more about postgraduates’ academic performance than their personal growth. The postgraduates wanted mentors to provide greater assistance in terms of their career path and development opportunities, which are insufficient in the existing professional relationship. The results validate previous studies indicating that mentors should play a proactive role in supporting postgraduates and helping them to achieve both academic goals and personal and professional aspirations [41]. Successful mentorship includes not only addressing curricular issues but also providing career opportunities for students [6].

Teachers’ characteristics are highly correlated with students’ academic achievement [42]. However, no evidence supports that a postgraduate academic or professional credential raises the quality of teaching [43]. Teachers rely on their postgraduate education to promote their personal development and professional career and to build their academic credentials [44]. The mentors accepted in postgraduate education had undergone long-term, formal professional training, could adequately apply innovations to the classroom environment, recognized different points of view on the education system, could discuss education applications with colleagues, and exhibited self-confidence in the workplace [44]. Additionally, postgraduate education enabled mentors to become experts in their subject, and they were more likely to be academically successful and popular with students. In contrast, mentors with bachelor’s degrees improved their personal abilities through self-learning. A lack of professional guidance makes mentors’ development more difficult. These mentors were eager to improve their educational competence, supportive competence and academic achievement in order to build good relationships with their students.

The literature regarding the relationship between students’ perceptions of good teaching and good instructors and their background characteristics is not extensive. Regarding sex, Anderson et al. [45], in their study among doctoral students, found that compared to men, women were more likely to endorse the traits of professional, expert and student-centered as characteristics of good instructors. Lavin et al. [46] and Korte et al. [47] found that compared to male students, female students tended to assign a higher rank to traits related to effective teaching. Our study is consistent with results reported in previous studies. Sex differences were found regarding mentors’ research competence, educational competence and communication competence, whereby female students assigned greater importance to all of these dimensions than did male students. The previous studies showed that female students were more likely than male students to suffer from anxiety and that the anxiety levels of female students were associated with their academic performance [48, 49]. As a result, female students might worry more about their study performance and, thus, value the competence of their mentors more than male students. More research is needed to further understand the reasons for these differences.

This study also has some limitations that should be considered. The study was limited to the Chinese system of pharmacy education, and the conclusions drawn from 8 universities may be enhanced by more participants and a longer study duration.

## Conclusions

**Fig. 2:**
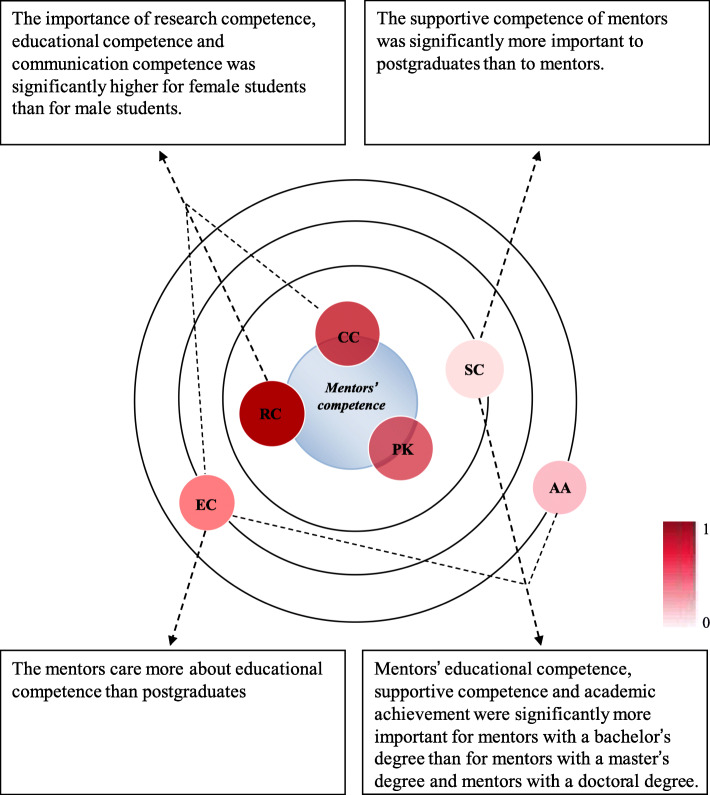
Schematic diagram of pharmacy mentor’s competence structure. The distance from the center of the circle represents the importance of each competence. The closer the competence is to the center, the more important it is. The darker the color of each competence, the greater the correlation coefficient with the research competence. RC = research competence, PK = professional knowledge, CC = communication competence, SC = supportive competence, EC = educational competence, AA = academic achievement

The main results of this study are illustrated in Fig. 2. We can draw the following conclusions: good mentors should possess three core competencies: research competence, professional knowledge and communication competence. These competencies are related rather than independent. The construction of a harmonious mentoring relationship should take full account of the student’s characteristics and expectations because graduate students care more about supportive competence and female students assign greater importance to mentors’ competence than male students. There should be more development opportunities for less educated mentors, as they have a greater need to increase their competence than more qualified mentors.

## Data Availability

The datasets used and analyzed in this study are available from the corresponding author upon reasonable request.
